# Patterns in root traits of woody species hosting arbuscular and ectomycorrhizas: implications for the evolution of belowground strategies

**DOI:** 10.1002/ece3.1147

**Published:** 2014-07-03

**Authors:** Louise H Comas, Hilary S Callahan, Peter E Midford

**Affiliations:** 1 Intercollege Graduate Program in Ecology, Department of Horticulture, The Pennsylvania State University103 Tyson Bldg, University Park, Pennsylvania, 16802; 2 USDA-ARS Water Management Research Unit2150 Centre Avenue, Bldg D Suite 320, Fort Collins, Colorado, 80526; 3 Department of Biological Sciences, Barnard College, Columbia University3009 Broadway, New York City, New York, 10027; 4 NESCent2024 W. Main Street, Suite A200, Durham, North Carolina, 27705

**Keywords:** Comparative method, *K* statistic, mycorrhizal colonization, nutrient acquisition strategies, root architecture, root branching intensity, root diameter, root morphology, root tissue density, specific root length

## Abstract

Root traits vary enormously among plant species but we have little understanding of how this variation affects their functioning. Of central interest is how root traits are related to plant resource acquisition strategies from soil. We examined root traits of 33 woody species from northeastern US forests that form two of the most common types of mutualisms with fungi, arbuscular mycorrhizas (AM) and ectomycorrhizas (EM). We examined root trait distribution with respect to plant phylogeny, quantifying the phylogenetic signal (*K* statistic) in fine root morphology and architecture, and used phylogenetically independent contrasts (PICs) to test whether taxa forming different mycorrhizal associations had different root traits. We found a pattern of species forming roots with thinner diameters as species diversified across time. Given moderate phylogenetic signals (*K *=* *0.44–0.68), we used PICs to examine traits variation among taxa forming AM or EM, revealing that hosts of AM were associated with lower branching intensity (*r*_PIC_ = −0.77) and thicker root diameter (*r*_PIC_ = −0.41). Because EM evolved relatively more recently and intermittently across plant phylogenies, significant differences in root traits and colonization between plants forming AM and EM imply linkages between the evolution of these biotic interactions and root traits and suggest a history of selection pressures, with trade-offs for supporting different types of associations. Finally, across plant hosts of both EM and AM, species with thinner root diameters and longer specific root length (SRL) had less colonization (*r*_PIC_ = 0.85, −0.87), suggesting constraints on colonization linked to the evolution of root morphology.

## Introduction

Ephemeral fine root tissues perform essential functions of absorbing nutrients and water. Yet, they remain one of the most poorly understood parts of plants in terrestrial ecology (Westoby and Wright 2006). Many studies of root traits have focused on root biomass allocation and proliferation rates (e.g., Hodge et al. 2009) with less investigation of root tissue morphology and physiology, which is critical for unraveling a variety of questions from organism-level questions of plant function in stressful or novel environments to ecosystem-level questions of plant functioning (Norby and Jackson 2000; McCormack et al. 2012; Reich 2014). From prior studies on root tissues traits, including those examining seedlings and adults, pot-grown and field-grown roots, we know that woody plants show particularly extensive variation in root structural and functional characteristics (e.g., Comas et al. 2002; Nicotra et al. 2002; Pregitzer et al. 2002; Withington et al. 2006; Comas and Eissenstat 2009; Paula and Pausas 2011). To date, our appreciation that roots of most woody species form essential associations with different types of mycorrhizal fungi for resource acquisition has yet to be fully integrated with what is known about patterns of root trait variation.

The evolution of root-like appendages was a critical component of the initial colonization of plants into a terrestrial environment over 400 Ma ago, with continued root modification likely allowing plants to further colonize and adapt to terrestrial landscapes (Remy et al. 1994; Algeo and Scheckler 1998; Heckman et al. 2001; Raven and Edwards 2001; Algeo and Scheckler 2010 and references within). The fossil record indicates that AM were the original type of mycorrhizas in vascular plants and predated the evolution of true roots (Remy et al. 1994; Heckman et al. 2001; but see Bidartondo et al. 2011). Among extant seed plant taxa, basal lineages are associated with coarse roots and less branching, in contrast to more recently diverged clades that more frequently have thin roots and greater branching intensity (Baylis 1972, 1975; Comas et al. 2012). The rise of EM associations in derived angiosperm and gymnosperm clades, especially beyond tropical latitudes, has been linked to further declines in CO_2_ and increased mineral weathering of soil during the Cretaceous, suggesting that the evolution of EM was a major event for the biosphere (Taylor et al. 2009; Comas et al. 2012). Mycorrhizal associations formed by plants have also been found to be concurrent with plant effects on carbon and nutrient cycling (Cornelissen et al. 2001; Phillips et al. 2013; but see Koele et al. 2012).

Among extant plants, strategies of more efficient nutrient acquisition and rapid growth are tied to species differences in root morphology and architecture (Comas et al. 2002; Comas and Eissenstat 2004, 2009). Root morphology governs total length or surface area per unit mass, directly impacting nutrient acquisition (Eissenstat 1991; Fitter 1991; Eissenstat and Yanai 1997). Root morphology is often summarized as the amount of root length per unit biomass (specific root length, SRL), which can be influenced both by root diameter and tissue density (Comas and Eissenstat 2004). However, SRL among woody species strongly correlates with root diameter with little correlation to variation in tissue density (Comas and Eissenstat 2004, 2009). Greater root branching also increases the efficiency of exploring the soil matrix (Fitter 1991). This reduced diffusion path of nutrients, and water to the root surface could increase plant hydraulic efficiency similar to that of increased vein density in leaves and petals (Boyce et al. 2009; Boyce and Leslie 2012). Plant roots, of course, also explore soil and acquire resources with assistance from their fungal partners.

Mycorrhizal symbioses in temperate forest communities are dominated by two forms: AM and EM. The evolution of EM, which are primarily distributed in temperate and boreal forests (Smith and Read 2008), has had many consequences, such as increasing plant access to different pools of soil nutrients, especially organically bound P and N, compared to AM (Finlay 2008 and references within). Structural differences between AM and EM have led to hypotheses of different selection forces driving root trait evolution of plants to form AM and EM (Brundrett 2002). In AM, fungi form associations within cortical cells along the root axis, such that thicker roots with a thick cortex may be able to support more AM per unit of root length or mass (Brundrett 2002; Guo et al. 2008b). In contrast, in EM, fungi predominately form Hartig nets in intercellular spaces of root tips, such that root systems with a higher frequency of tips may be able to support more EM (Brundrett 2002). Neither of these hypotheses have been fully explored by analyzing variation among species in a phylogenetic framework.

Additionally, a hypothesized general trade-off between root morphological traits and dependency on mycorrhizas has been suggested anecdotally based on observations in species with AM habit that fine-rooted species support less colonization and potentially benefit less from colonization than coarse-rooted species (St. John 1980; Graham and Syvertsen 1985; Reinhardt and Miller 1990; Manjunath and Habte 1991; Hetrick et al. 1992). Thin roots with higher SRL may generally be less dependent on mycorrhizas if their morphology is better suited for nutrient acquisition without mycorrhizas (Baylis 1975; Brundrett 2002), although exceptions can be found (Siqueira and Saggin-Junior 2001). A broader test of morphological trade-offs in root tissues with mycorrhizal colonization requires investigating diverse additional plant taxa, including EM hosts.

A central goal of this study was to determine whether there is systematic variation across plant clades and among taxa with contrasting mycorrhizal habits and nutrient acquisition strategies (Brundrett 2002; Smith and Read 2008). Root trait variation was previously examined in 25 species from this ecosystem to identity major axes and broad patterns of variation, primarily using multivariate analyses to identify and quantify traits that varied (Comas and Eissenstat 2009). Here, we broadened sampling to focus on variation among different mycorrhizal plant hosts along a broad taxonomic range with phylogenetic analyses to address questions of trait adaptation. We examined root traits of 33 woody species collected from two different forests at the intersecting range of temperate and boreal species. Because site-to-site variation in soil and other environmental factors can have large effects on root traits, a strength of working with coexisting species is maintaining a degree of consistency in environmental and soil factors. However, such an approach introduces the possibility that site factors can ecologically filter the species composition of a community, limiting the taxonomic scope of the analysis. Acknowledging these strengths and weaknesses, we proceeded with our analysis, aiming to more fully explain patterns in the large variation in fine root traits among coexisting northeastern US woody species that was documented in previous research (Comas and Eissenstat 2009). Using a large species list and broad phylogenetic coverage, we quantified the phylogenetic signal of fine root traits (branching intensity, SRL, diameter, and tissue density). We then explored trait differences between plants forming AM and EM to ask “*are there differences in root morphology between plants forming AM and EM independent of their shared ancestry?*” We specifically assessed the hypotheses that (1) there would be phylogenetic signals in root traits and (2) roots of AM plants would generally have less branching intensity and (3) less SRL than EM plants with (4) differences in SRL between AM and EM due to thicker diameter rather than greater tissue density of AM. Finally, we assessed the hypothesis that species with smaller root diameter, longer SRL, greater density, and more branching would have less mycorrhizal colonization.

## Methods

### Species and sampling locations

**Figure 1:**
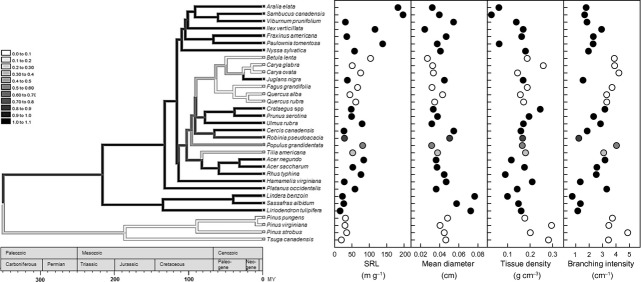
Phylogenetic relationships of the 33 species and their root traits. Branch color in phylogenetic tree indicates category of mycorrhizal habit formed along a numerical index from 0 (white, exclusively EM) to 1 (black, exclusively AM) (Table 1). Branch lengths of tree indicate proximity of relationships in units of million years. Plotted next to tree are species averages for specific root length (SRL), mean root diameter, root tissue density, and root branching intensity.

Thirty-three woody species common to temperate northeastern US mesic forests were sampled (Table 1; Fig. 1). Clades spanned Pinaceae, magnoliids, core eudicots, eurosids I and II, core asterids, and euasterids I and II and included species with different plant growth strategies, such as fast- and slow-growing habits, different shade tolerance and leaf longevity, as well as different mycorrhizal formations (see Table 1) (Comas and Eissenstat 2004, 2009). Twelve of the species included in this study predominantly hosted EM and 21 predominantly hosted AM (Harley and Harley 1987; Wang and Qiu 2006; personal observation; and screening published plant lists for errors, sensu Brundrett 2009). Mycorrhizal types of seven species not found in the literature were determined from field samples as described below. Categories of AM- and EM-forming plant species were assigned numerical index values according to the frequency of root colonization by different types of mycorrhizal fungi found, between 1 for exclusively AM and 0 for exclusively EM (Table 1).

**Table 1:** Species examined in this study and the mycorrhizas formed by each.

Species	Common name	Mycorrhizas formed	Myc. cat.
*Betula lenta* L.	Sweet birch	EM	0.00
*Carya glabra* (Mill.) Sweet	Pignut hickory	EM	0.00
*Carya ovata* (Mill.) K. Koch	Shagbark hickory	EM	0.00
*Fagus grandifolia* Ehrh.	American beech	EM	0.00
*Pinus pungens* Lamb.	Mountain pine	Predominately EM, form AM on rare occasion	0.05
*Pinus strobus* L.	White pine	Predominately EM, form AM on rare occasion	0.05
*Pinus virginiana* Mill.	Virginia pine	Predominately EM, form AM on rare occasion	0.05
*Quercus alba* L.	White oak	Predominately EM, form AM occasionally	0.10
*Quercus rubra* L.	Red oak	Predominately EM, form AM occasionally	0.10
*Tsuga canadensis* (L.) Carrière	Eastern hemlock	Predominately EM, form AM occasionally	0.10
*Tilia americana L*.	American basswood	Mostly EM, form AM occasionally	0.20
*Populus grandidentata* Michx.	Bigtooth aspen	Frequently both EM and AM, benefits most from EM	0.40
*Robinia pseudoacacia* L.	Black locust	Mostly AM but frequently EM	0.75
*Acer negundo* L.	Boxelder	Predominately AM, form EM occasionally	0.90
*Acer saccharum* Marsh.	Sugar maple	Predominately AM, form EM occasionally	0.90
*Prunus serotina* Ehrh.	Black cherry	Predominately AM, form EM occasionally	0.90
*Ulmus rubra* Muhl.	Slippery elm	Predominately AM, form EM occasionally	0.90
*Crataegus spp*^1^	Hawthorn	Predominately AM, form EM on rare occasion^2^	0.95
*Fraxinus americana* L.	White ash	Predominately AM, form EM on rare occasion	0.95
*Ilex verticillata* (L.) A. Gray	Winterberry holly	Predominately AM, form EM on rare occasion^2^	0.95
*Juglans nigra* L.	Black walnut	Predominately AM, form EM on rare occasion	0.95
*Sambucus nigra* L. ssp. *canadensis* (L.) R. Bolli	American elderberry	Predominately AM, form EM on rare occasion^2^	0.95
*Aralia elata* (Miq.) Seem.	Japanese angelica tree	AM^3^	1.00
*Cercis canadensis* L.	Redbud	AM^3^	1.00
*Hamamelis virginiana* L.	Witch hazel	AM^3^	1.00
*Lindera benzoin* (L.) Blume	Spicebush	AM	1.00
*Liriodendron tulipifera* L.	Tuliptree	AM	1.00
*Nyssa sylvatica* Marsh.	Black gum	AM^3^	1.00
*Paulownia tomentosa* (Thunb.) Siebold & Zucc. ex Steud.	Princess tree	AM^3^	1.00
*Platanus occidentalis* L.	American sycamore	AM^3^	1.00
*Rhus typhina* L.	Staghorn sumac	AM^3^	1.00
*Sassafras albidum* (Nutt.) Nees	Sassafras	AM	1.00
*Viburnum prunifolium* L.	Blackhaw viburnum	AM	1.00

Categories of mycorrhizal types were assigned from observations in the literature, with screening to avoid erroneous citations (Harley and Harley 1987; Grange et al. 1997; Dickie et al. 2001; Wang and Qiu 2006; Quoreshi and Khasa 2008). Mycorrhizal categories for species not found in the literature were based on our field observations as noted.

1 Probably *Crataegus pennsylvanica* Ashe but readily hybridizes with other *Crataegus* species.

2 Suspicious observations of EM colonization.

3 From field observations during this study.

Root sampling was performed in three different years. In spring 1999, 25 species were sampled from two stands in the Penn State Stone Valley Experimental Forest (Barree Township, Huntingdon County, PA) in relatively low-lying areas adjacent to a stream. Both were approximately 65 years old, even-aged, and predominantly hardwoods. Soil in one was an Ernest silt loam (Fine–loamy, mixed, superactive, mesic Aquic Fragiudult) and in the other a Newark silt loam (Fine–silty, mixed, active, nonacid, mesic Aeric Fluvaquent). All measurements were taken from individuals that were either in the upper canopy or open grown, with diameter breast height ranging from 7 to 75 cm. All species were sampled from both stands, except for *Lindera benzoin*, which was only found in one stand.

In spring 2008, eight new species, as well as 16 of the original 25, were sampled from Black Rock Forest (BRF) in the town of Cornwall, located in Orange County, New York, USA, and a different section of the Penn State Forest than previously sampled in 1999. Soil at BRF was a Swartswood and Mardin very stony soil, characteristically gravelly loam to gravelly silt loam. Soil in this section of the Penn State Forest was an Atkins or Andover fine loam. As a stream also traversed these sites, individual plants sampled were similar in placement and size to those previously sampled. Of the species sampled, all were sampled from both sites, except for *Sambucus nigra, Aralia elata*, and *Paulownia tomentosa* that were only sampled in New York, and *Pinus strobus, Robinia pseudoacacia, Prunus serotina, Juglans nigra, Ilex verticillata*, and *S. alba* that were only sampled in Pennsylvania, because they were not present at both sites.

Eight species (*Acer negundo*, *Acer saccharum*, *Betula lenta*, *Carya glabra*, *Carya ovata*, *Fagus grandifolia*, *Quercus alba*, and *Quercus rubra*) were sampled in 2000 from the same two stands sampled in the Penn State Forest sampled in 1999 but with root bag techniques (described below) to obtain root tissues of similar ages for assessing mycorrhizal colonization levels (Comas and Eissenstat 2004).

### Tissue sampling and processing

In 1999, samples were taken from undisturbed soil as described below (Comas and Eissenstat 2009). Trait assessment of roots growing in their natural condition incorporates the range of intraspecific variation, including effects of a natural range of mycorrhizal colonization. Samples were collected from three plants of each species over 6 weeks in June and July (Comas and Eissenstat 2009). Because one stand was larger than the other, two plants were sampled at the larger stand and one at the smaller stand. Six of the 75 root samples collected were omitted from analysis because roots had many dry sections that hindered processing and trait quantification. In 2008, between the two sites, samples were taken from an average of 4.9 plants per species (1–9 plants depending on their presence and frequency in the community). Samples were collected in July within 1 week in Pennsylvania and then during the following week in New York. Eight of the 117 samples collected were omitted from final analyses because they were partially desiccated.

**Figure 2:**
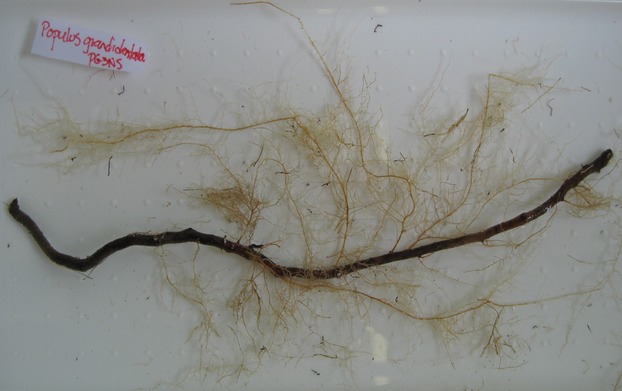
Populus grandidentata roots during processing. Fine root clusters were left attached to higher-order roots until samples were processed. A processed sample consisted of multiple clusters of first- and second-order roots (root tips being 1st order) collected from the same tree.

Sampling in undisturbed soil involved excavating roots from the top 20 cm of soil and tracing intact root clusters back to the woody root system and then back to the trunk for species identification. Fine nonwoody roots were left attached to woody roots, sprayed with water, and kept in plastic bags refrigerated prior to washing with tap water (Fig. 2). Only the distal two branch orders were taken as a cluster because mounting evidence suggests that delineating fine roots in this manner most narrowly defines them as the nutrient-acquiring, ephemeral portion of a woody root system (Xia et al. 2010). As each cluster was collected, it was placed in distilled water until a pool of clusters was accumulated. Additionally, roots in 2008 were stained in neutral red (0.16 mg/L) prior to imaging for further contrast.

Samples were spread in water and imaged with a desktop scanner and transparency adapter. In 1999, WinRhizo software (Quebec, Canada) was used to acquire and analyze images with automatic brightness and contrast settings in transparency mode (Comas and Eissenstat 2009). In 2008, HP Scanjet 8300 software was used, also with automatic brightness and contrast settings in transparency mode, and Delta-T SCAN software (Cambridge, UK) was used for image analysis. No difference has been found in root traits assessed by these protocols and software (Bouma et al. 2000). After imaging, samples were oven dried for 2 days, equilibrated to room temperature on desiccant, and weighed. Analysis software generated estimated length, average and modal diameter, volume, and tip counts of roots in each image. Specific root length was calculated from the length divided by mass, and tissue density from mass divided by estimated volume. Branching intensity was calculated from the number of tips divided by length. This simple measure could be used to quantify branching intensity because only the two most terminal branches were analyzed.

In 2000, roots collected were trained to grow in bags. When analyzing data of roots sampled from root bags, our focus was to evaluate root traits associated with variation in mycorrhizal colonization. An advantage of this sampling approach is its ability to control for variation associated with root age, and its potential impact on mycorrhizal colonization and root traits (Resendes et al. 2008; Lee et al. 2014). While disturbance during installation may have impacted mycorrhizal colonization, this method controlled for root age effects on colonization with any potential methodological impact on colonization and root traits uniformly spread across all species. Root growth bags were constructed from porous nylon landscape fabric as 23 × 20 cm sacks sewn on three sides and installed under two trees in each of two stands (four total) for each species in early May 2000 (Comas and Eissenstat 2004). Bags were filled with a 1:3 mixture (v:v) of quartz sand and sieved field soil using soil collected at the spot of each installation. Differences in root morphological traits have been shown to be consistent among several of these species across different soil media (Comas et al. 2002; Comas and Eissenstat 2004). A woody root >4 mm in diameter was planted in each bag after tracing it back to the tree for identification and removing all fine roots. Fine root fragments chopped into <1-cm segments were mixed with soil in the bag as a source of fungal inoculum. At harvest, 5–6 weeks later, the woody root “planted” in the bag was cut and the entire bag was excavated. Bags were brought to the laboratory intact for washing and processing with WinRhizo as described above.

The same root samples used for morphological assessment were also examined for mycorrhizal colonization, which required rehydration. Roots were rehydrated concurrently with clearing either by soaking in 10% KOH for 16–36 h at room temperature or boiling at 90°C for 10 min. For roots sampled in 2008 that were stained with neutral red, roots were then rinsed with 85% ethanol followed by an 85% ethanol soak for 5 min to remove neutral red. Although enough of the neutral red was removed to allow for examination of colonization, staining with neutral red is not recommended in future work if roots are to be processed for mycorrhizal colonization. Samples were soaked for 15 min in either 3% or 30% H_2_O_2_ as needed to remove plant pigments and then washed with water and checked under the dissecting microscope for cortical cell layer visibility. Roots were acidified with 5% hydrochloric acid (HCl) for 5 min and incubated in 0.05% trypan blue stain in a 90°C water bath for 20 min. The roots were rinsed with water and stored in lactoglycerol (1:1:1 lactic acid, glycerol, and water) (Grace and Stribley 1991; Comas and Eissenstat 2004). Each sample was mounted on slides with glycerin jelly (Widden 2001). Samples were examined for AM with a magnification of 200 × and for EM under a dissecting microscope (15×). Colonization of AM was scored using the magnified intersections method (Giovannetti and Mosse 1980; McGonigle et al. 1990). Arbuscules, vesicles, and nonseptate hyphae within roots were scored as colonization and then expressed as percent of total segments scored. Colonization of EM was scored if a tip appeared sheath covered and expressed as percent of total root tips scored.

### Statistical analyses

Analyses of phylogenetic trait patterns were conducted on species trait means of data across all years available for each species. Prior analyses found that trait variation among species outweighed variation within species (Comas and Eissenstat 2009). Branching intensity was found to differ for species between the 1999 and 2008 data set, likely due to differences in the age of root clusters between the data sets because spring conditions led to delayed tree growth in the 2008 growing season. Thus, a correction factor determined through linear regression (not shown) was applied to normalize the 2008 data prior to averaging means by species.

A phylogenetic tree was assembled from current evaluations of plant species diversification that used recent methods, including relaxed-clock analyses and fossil-based calibrations, to date species divergences (Savolainen et al. 2000; Wikstrom et al. 2001; Angiosperm Phylogeny Group 2003; Magallon and Sanderson 2005; Gernandt et al. 2008; Smith et al. 2010) (Fig. 1). There were no polytomies in the tree. Branch lengths were estimated with maximum likelihood methods.

The presence of phylogenetic signal in traits was tested using the randomization procedure of Blomberg et al. (2003) and the magnitude assessed with the *K* statistic (Blomberg et al. 2003), with values varying from 0 (no signal) to infinity. Both tests were performed using the phylosignal procedure in the R package Picante (Kembel et al. 2010).

To further test the presence and fit of the phylogenetic signal, the fitContinuous procedure in the R package Geiger (Harmon et al. 2009) was used to fit three models of continuous character evolution on the distribution of root traits. In contrast to the white model of no phylogenetic signal, we fit models for evaluating evidence for stabilizing selection (OU = Ornstein-Uhlenbeck for a random walk of traits with a central tendency) and for trait evolution toward randomly fluctuating selective optima (BM = Brownian Motion).

Correlations between root traits and mycorrhizal types for 33 terminal taxa, and between root traits and mycorrhizal colonization of roots for eight terminal taxa, were assessed with phylogenetically independent contrasts (PICs) (Felsenstein 1985) to account for phylogenetic sampling of species and trait correlation along evolutionary time frames. PICs have been found to give robust estimates of the correlation between characters with as few as eight terminal taxa (Oakley and Cunningham 2000). PIC analyses were computed with the PDAP:PDTREE Package version 1.15 (Midford et al. 2010) within the Mesquite system (Maddison and Maddison 2010) for phylogenetic computing using the fully resolved phylogenetic tree described above. PDAP was also used to test assumptions of the independence of contrasts and their associated standard deviations (square root of sum of squared branch lengths) (Garland et al. 1991), which were met in all cases.

## Results

### Phylogenetic pattern in root traits

The *K* statistic indicated moderate phylogenetic signal in SRL, root diameter, and branching intensity among the 33 species examined and no signal in tissue density (Table 2). More recently, divergent clades had a greater percentage of species with thinner root diameters, greater SRL and increased branching intensity, with a few species persisting that had relatively thick root diameters, and low SRL and branching (Fig. 1). Furthermore, the phylogenetic distribution of root diameter suggested a general pattern of more recently diverged species having thinner roots than older divergences (correlation between reconstructed root diameter at nodes and the distance of each node from the root of the phylogenetic tree: *r *=* *0.29, *P *=* *0.11).

**Table 2:** Phylogenetic signal in four root traits describing fine root morphology and architecture of ephemeral clusters.

Trait	*K*	*P*-value	Difference from lowest AIC value		
			White	OU	BM
SRL	0.50	0.017	483.3	**0.0** *(0.0078)*	**1.9**
Mean diameter	0.68	0.004	8.0	**0.0** *(0.0046)*	**1.0**
Tissue density	0.21	0.687	**0.0**	**1.9** *(0.207)*	26.5
Branch Intensity	0.44	0.002	4.1	**0.0** *(0.0055)*	**1.1**

Signal strength was assessed by the *K* statistic and its *P*-value. Fit was assessed with likelihood models for continuous character evolution (OU = Ornstein-Uhlenbeck for trait evolution with stabilizing selection; BM = Brownian motion as trait evolution toward randomly fluctuating optima; and white = white noise for no phylogenetic signal), with the best model identified by the lowest Akaike information criterion (AIC) value. Models with AIC values within 1–2 of the minimum value (bold-faced) have enough support to warrant consideration, those with values 4–7 more than the minimum receiving considerably less support, and those with values >10 essentially unsupported. For the OU model, the *s* parameter appears in parentheses and italic next to the AIC value.

Models of character evolution indicated similar patterns of phylogenetic signal among root traits. The white noise model (no phylogenetic signal) was ill-fitting for SRL, root diameter, and branching intensity, indicating that there was signal in these traits (Table 2). For tissue density, consistent with the randomization test indicating no significant phylogenetic signal (*K* statistic), the white noise model was one of two models that best fit the trait distribution (Table 2). For SRL, root diameter, and branching intensity, the OU model was generally best fitting, indicating the operation of stabilizing selection in these traits, but the BM model, in which PIC analyses are based on, was also close, indicating that PIC analyses are appropriate for this data.

### Root traits and mycorrhizal types

**Figure 3:**
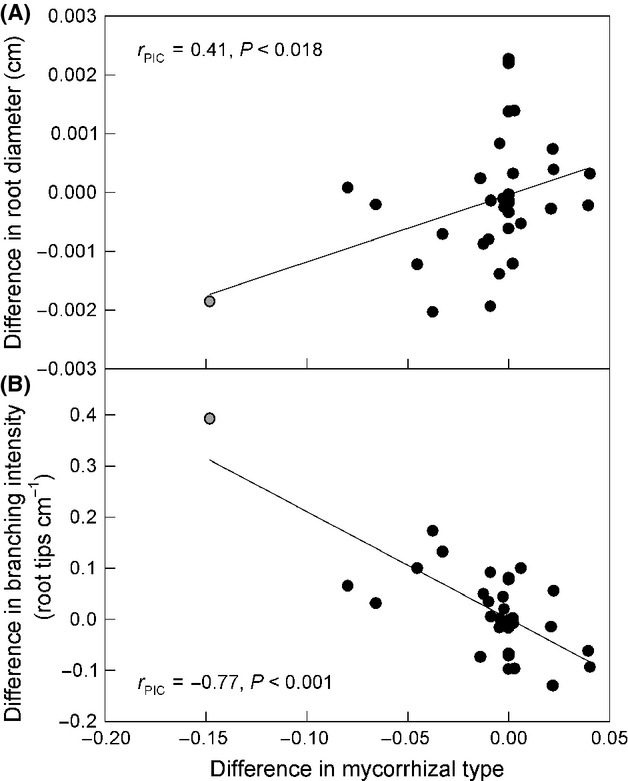
Relationships between mycorrhizal and root trait adaptations. Scatterplots show the relationship between mycorrhizal types and mean root diameter (A) and root branching intensity (B) at tree nodes from the analysis of phylogenetically independent contrasts (PICs). Thirty-two contrasts were assessed among the 33 species examined. Phylogenetic branch lengths among species were accounted for, estimated with maximum likelihood methods from the recent literature. Mycorrhizal associations formed by each plant species are given in Table 1. Exclusive AM were scored as 1 and EM as 0. Species forming predominately AM had thicker roots and less branched root clusters than those forming EM. Trait relationships were similar without *Juglans nigra* (gray circle), although less strong for root diameter (*r*_PIC_ = 0.27, *P *=* *0.14 and *r*_PIC_ = −0.56, *P *<* *0.001, respectively).

We found that plants forming EM and AM were associated with different root architecture and morphology (Fig. 3). Specifically, we found that hosts of EM had greater root branching intensity and thinner root diameter than hosts of AM, but there is wide variation within both categories. Trait relationships were similar even with the exclusion of *Juglans nigra*, a plant forming AM that has close relatives hosting EM (Fig. 3). There was no relationship between root tissue density and mycorrhizal type (*r*_PIC_ = 0.14; *P *=* *0.42), nor between SRL and mycorrhizal type (*r*_PIC_ = 0.16; *P *=* *0.37).

### Root traits and mycorrhizal colonization levels

**Figure 4:**
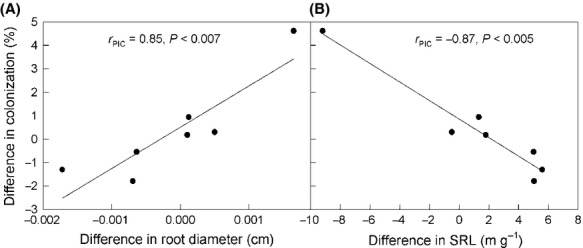
Relationships between mycorrhizal colonization and root traits. Scatterplots show the relationship between mycorrhizal colonization (percentage of total root tips colonized by fungi for EM or cortical cells colonized by fungi for AM) and root diameter (A), and specific root length (SRL, m g^−1^) (B) at tree nodes from the analysis of phylogenetically independent contrasts (PICs). Seven contrasts were assessed among the 8 species for which data were available. Phylogenetic branch lengths among species were accounted for, estimated with maximum likelihood methods from the recent literature.

Among the eight species and seven contrasts for which colonization data were available, mycorrhizal colonization was significantly higher in species with thicker fine root clusters and lower in those with greater SRL (Fig. 4). There were no significant correlations between colonization levels and traits of root tissue density or branching intensity (*r*_PIC_ = 0.10, *P *=* *0.82; *r*_PIC_ = −0.05, *P *=* *0.92).

## Discussion

We found moderate phylogenetic signals in root diameter, SRL, and branching intensity, signifying a tendency of trait similarity among closely related species (sensu Blomberg and Garland 2002). We found a general overall pattern of root diameter becoming thinner as species diversified, similar to the pattern found in a data set of tropical and subtropical species (Comas et al. 2012). No phylogenetic signal was found in tissue density, which other studies of temperate woody species have suggested may vary more in response to soil microsite properties rather than in association with differences among species (e.g., Comas and Eissenstat 2009). After accounting for phylogenetic signals present, we found root traits differed between plant hosts forming EM and AM, with hosts of EM having thinner roots and greater branching intensity. Consistent with our hypotheses, these trait differences suggest either that EM placed different trait selection pressures than AM as plants adapted to forming these associations, or that plants with different traits were predisposed to evolving different mycorrhizal associations. Finally, lower mycorrhizal colonization levels were also correlated with thinner roots and longer SRL after accounting for phylogenetic signals, suggesting trade-offs between colonization levels and root morphology linked to the evolution of these traits.

In general, phylogenetic signals can be caused by different processes. Similarity in root traits among related species may reflect phylogenetic inertia (i.e., the origin of similar traits among relatives), and/or evolution by Brownian motion (i.e.*,* random genetic drift along a hierarchical phylogeny), while adaptation to environmental conditions via natural selection can decrease phylogenetic signals (Blomberg and Garland 2002). While potentially interesting to separate effects of different processes, the moderate yet significant phylogenetic signal found in the analyses presented here for root diameter, SRL, and branching intensity suggest that both phylogenetic inertia and adaptation shape contemporary trait variation in these traits (sensu Blomberg and Garland 2002; Powell et al. 2009). Our analyses of phylogenetic signals showed limited support for purely random models (no phylogenetic signal) explaining these trait patterns. Analyses suggested that the OU model and, thus, stabilizing selection of traits best fit the data, but the BM model and thus randomly fluctuating optima warranted consideration. It is possible that a combination of directional and diversifying selection has operated on these traits through time.

Directional change in root trait evolution has long been hypothesized. Initial hypotheses of plants evolving thinner roots over time were proposed based on limited examples of species contrasts between plants adapted to different environmental conditions (e.g.*,* Magnolias and grasses) (Baylis 1972, 1975). Evidence of plants evolving thinner roots with more branching across evolutionary time frames is found in the fossil record of early land plants (Algeo and Scheckler 1998). Evolutionary root trait and mycorrhizal developments may explain the increase in biological weathering and gradual decline in CO_2_ during the Cretaceous coinciding with the rise of angiosperms (Taylor et al. 2009; Comas et al. 2012). Clear evolutionary patterns are found of fine roots becoming thinner as angiosperms radiated, as compared to remaining relatively coarser in more basal and less diverse angiosperm and nonangiosoperm lineages (e.g., cycads, gnetophytes, and gingko) (Comas et al. 2012), similar to patterns here and elsewhere (Chen et al. 2013). Notably, 140 Ma of Cretaceous angiosperm diversification resulted in SRL spanning a 20-fold increase (Comas et al. 2012). The data presented here and elsewhere show a wide range of variation in SRL even among coexisting northeastern US species, fitting within that scope of variation (Comas and Eissenstat 2009). Trait variation here and elsewhere also appears to increase rather than remain narrow and stable, such that recently diverged lineages include species with both conserved (thicker root diameters, and less SRL and branching intensity) and derived traits (thinner root diameters, and greater SRL and branching intensity) compared to basal lineages (Comas et al. 2012).

EM appears to have arisen multiple times in different host lineages, based on the analysis of the distribution of EM among plant hosts (Fitter and Moyersoen 1996; Brundrett 2009; Fig. 1). Our analyses cannot eliminate the possibility that root traits adapted for other reasons, predisposing plants to evolve the type of mycorrhizal symbioses that they currently form. Our finding of significantly greater root branching intensity in hosts of EM is merely congruent with adaptation to accommodate more EM because root tips are the main site of colonization (Massicotte et al. 1989; Smith and Read 2008). Similarly, our finding hosts of EM associated with thinner root diameters than hosts of AM is congruent with selection favoring hosts of AM to produce a thicker cortex for supporting more extensive colonization by mycorrhizal fungi, in contrast to hosts of EM whose colonization is likely to be independent of cortical thickness (Brundrett 2002). Root diameters of EM reported here include fungal mantles on root tips, which can add 0.03–0.1 mm thickness or approximately 7.5% to the cross-sectional area of these root tips (Withington et al. 2006). This overestimated thickness of EM may underrate the total root diameter difference between hosts of AM and EM, such that the contrasts found here may be conservative. Correlations between mycorrhizal colonization levels and root morphology analyzed here, especially with the addition of species hosting EM, expand on previous investigations of the effects of root morphology on colonization. Formations of EM are independent of root diameter and SRL; our correlations of colonization levels with root morphology of species forming EM suggests that benefits in soil resource acquisition by thinner roots with greater SRL may be enough to drive root trait change without also serving to limit root colonization as would occur in AM.

Consideration of root traits with and without mycorrhizas raises the important issue of whether root traits *per se* display responsiveness (i.e., phenotypic plasticity) to colonization by mycorrhizal fungi (Schroeder and Janos 2005). Indeed, signaling molecules such as strigalactones released by fungi forming AM are known to bind to receptors in plant root cells after roots are formed to regulate cell wall remodeling to host AM (Parniske 2008), but the role of this signaling in evoking predictable and consistent plasticity in tissue-level traits such as root morphology and architecture is less clear-cut (Lee et al. 2014). Documented plasticity in root traits to colonization has varied among plant species in degree and direction (Schroeder and Janos 2005 and references within). The natural and common root phenotype of most species is to be colonized with diverse fungi, generally of one type, that vary in their functioning and specificity and potential effects on root morphology (Helgason et al. 2002). However, greenhouse studies with several of the plant species examined here, colonized with diverse field-collected inoculum, found similar among-species differences among colonized and uncolonized root samples and no evidence of mycorrhizal colonization eliciting consistent patterns of plasticity in root diameter or branching intensity, suggesting that root trait differences among species are greater than effects of colonization (Comas et al. 2002; Comas and Eissenstat 2004; Lee et al. 2014).

In summary, investigations of belowground strategies are of particular interest for woody vegetation, which have a dynamic fine root system for the exploration of soil resources (Eissenstat and Yanai 1997; Pregitzer et al. 2002; Guo et al. 2008a). Examinations of phylogenetic signals and root trait variation among plants with different strategies for resource acquisition, such as AM and EM, are important first steps in exploring evolutionary and ecological hypotheses of resource acquisition strategies. Our results implore further field investigation and suggest potentially interesting evolutionary and ecological hypotheses about selection favoring root traits and their plasticity, and relating these traits to nutrient foraging efficiency with and without mycorrhizas, the mechanism through which selection might occur (Brundrett 2002). Patterns of species variation in root traits among the species examined here provided some clues to selection pressures that have shaped roots and development of terrestrial ecosystems, although investigation of species from other biomes are needed to test the generality of these changes in root morphology. In this study, we focused on community-level patterns of root trait diversity in Northeastern temperate mesic forests, which have diverse coexisting woody species with large variation in root morphology and different mycorrhizal types adapted to similar environmental conditions. To explore convergent patterns of root trait adaptations, we will need to examine root traits of species in more biomes and taxonomic clades.

## Supporting Information

**Appendix S1.** Species trait averages.
