# Supplementary material for: Patterns in root traits of woody species hosting arbuscular and ectomycorrhizas: implications for the evolution of belowground strategies
Source: Ecol Evol. 2014 Jul 3;4(15):2979–90. doi: 10.1002/ece3.1147 (PMC4161172; doi:10.1002/ece3.1147)
Supplement: Supplementary file 1 — Appendix S1. Species trait averages. [file ece30004-2979-sd1.docx]

**Appendix 1. Species trait averages.** Traits include specific root length (SRL), mean root diameter, root tissue density and root branching intensity. For each species, trait values were first averaged by site and year and then by species.

|  |  |  |  |  |
| --- | --- | --- | --- | --- |
| **Species** | **SRL** | **Mean diam.** | **Tissue density** | **Branching intensity** |
|  | **m g^-1^** | **cm** | **g cm^-3^** | **cm^-1^** |
| *Acer negundo* | 83.8 | 0.036 | 0.117 | 3.18 |
| *Acer saccharum* | 52.4 | 0.037 | 0.175 | 2.57 |
| *Aralia elata* | 182.5 | 0.032 | 0.061 | 1.78 |
| *Betula lenta* | 103.5 | 0.027 | 0.187 | 3.87 |
| *Carya glabra* | 50.8 | 0.033 | 0.259 | 3.89 |
| *Carya ovata* | 75.0 | 0.033 | 0.144 | 4.22 |
| *Cercis canadensis* | 27.1 | 0.055 | 0.159 | 1.85 |
| *Crataegus spp* | 47.9 | 0.033 | 0.245 | 3.17 |
| *Fagus grandifolia* | 66.3 | 0.032 | 0.188 | 3.70 |
| *Fraxinus americana* | 35.1 | 0.047 | 0.163 | 2.31 |
| *Hamamelis virginiana* | 27.8 | 0.047 | 0.209 | 1.34 |
| *Ilex verticillata* | 116.4 | 0.024 | 0.169 | 2.92 |
| *Juglans nigra* | 36.8 | 0.045 | 0.169 | 1.54 |
| *Lindera benzoin* | 22.6 | 0.077 | 0.100 | 0.74 |
| *Liriodendron tulipifera* | 15.9 | 0.072 | 0.160 | 1.15 |
| *Nyssa sylvatica* | 57.4 | 0.041 | 0.180 | 1.95 |
| *Paulownia tomentosa* | 138.3 | 0.037 | 0.062 | 2.29 |
| *Pinus pungens* | 31.4 | 0.048 | 0.176 | 3.73 |
| *Pinus strobus* | 34.9 | 0.045 | 0.201 | 4.87 |
| *Pinus virginiana* | 29.9 | 0.040 | 0.295 | 3.47 |
| *Platanus occidentalis* | 58.7 | 0.036 | 0.143 | 3.31 |
| *Populus grandidentata* | 80.8 | 0.032 | 0.165 | 4.04 |
| *Prunus serotina* | 48.9 | 0.038 | 0.195 | 2.33 |
| *Quercus alba* | 44.1 | 0.043 | 0.157 | 3.30 |
| *Quercus rubra* | 60.9 | 0.035 | 0.171 | 3.31 |
| *Rhus typhina* | 75.9 | 0.045 | 0.090 | 2.53 |
| *Robinia pseudoacacia* | 28.5 | 0.050 | 0.167 | 1.23 |
| *Sambucus nigra* | 197.0 | 0.040 | 0.028 | 1.66 |
| *Sassafras albidum* | 26.5 | 0.058 | 0.148 | 1.35 |
| *Tilia americana* | 52.0 | 0.038 | 0.180 | 3.08 |
| *Tsuga canadensis* | 19.6 | 0.046 | 0.282 | 3.44 |
| *Ulmus rubra* | 79.2 | 0.032 | 0.170 | 2.84 |
| *Viburnum prunifolium* | 31.2 | 0.055 | 0.139 | 1.84 |
